# Supplementary material for: Triggered chain reaction: The meanings of symptom clusters for patients with chronic obstructive pulmonary disease: A cross-sectional qualitative study
Source: PLoS One. 2026 May 4;21(5):e0348370. doi: 10.1371/journal.pone.0348370 (PMC13138636; doi:10.1371/journal.pone.0348370)
Supplement: S2 File — (DOCX) [file pone.0348370.s002.docx]

**S2 File. Summary of methodological approaches and their role in this study**

**Study design:** A descriptive qualitative study

**To promote explicit and comprehensive reporting of qualitative studies:** (COREQ) checklist

**Theoretical frameworks:** Theory of Unpleasant Symptoms (TOUS) and Theory of "biographical disruption"

**Data collection methods:** Face-to-face, semi-structured interviews

**Analytical approach:** Framework approach

**Quality criteria fit together:** Realist approach
