# Supplementary material for: Triggered chain reaction: The meanings of symptom clusters for patients with chronic obstructive pulmonary disease: A cross-sectional qualitative study
Source: PLoS One. 2026 May 4;21(5):e0348370. doi: 10.1371/journal.pone.0348370 (PMC13138636; doi:10.1371/journal.pone.0348370)
Supplement: Attachment — Submitted filename: renamed_fba9a.docx [file pone.0348370.s004.docx]

^30th^ March 2026

Taiwo Opeyemi Aremu,

**Re: Triggered chain reaction: The meanings of symptom clusters for patients with chronic obstructive pulmonary disease: A cross-sectional qualitative study**

We wish to express our gratitude to the editor and reviewers for taking the time to read our manuscript submitted to PLOS One. They have proposed a number of important modifications to the manuscript to improve its content that we have now addressed with our resubmission. In this revised version, changes to our manuscript within the document are all highlighted by using red coloured text. Our point-by-point responses to the editor and reviewers’ comments are listed below this letter.

| **Academic Editor** | **Response to Editor** |
| --- | --- |
| 1. Please ensure that your manuscript meets PLOS ONE's style requirements, including those for file naming. The PLOS ONE style templates can be found at https://journals.plos.org/plosone/s/file?id=wjVg/PLOSOne_formatting_sample_main_body.pdf and  https://journals.plos.org/plosone/s/file?id=ba62/PLOSOne_formatting_sample_title_authors_affiliations.pdf | We thank the editor for pointing out this important issue.  We had thoroughly reviewed the provided style templates and had updated our manuscript to ensure full compliance. |
| 2.We noticed you have some minor occurrence of overlapping text with the following previous publication(s), which needs to be addressed:  - DOI: 10.1371/journal.pone.0265861  - http://dx.doi.org/10.1016/j.ejon.2014.02.004 1462-3889/  In your revision ensure you cite all your sources (including your own works), and quote or rephrase any duplicated text outside the methods section. Further consideration is dependent on these concerns being addressed. | We have carefully reviewed the entire manuscript against the cited publications  (DOI: 10.1371/journal.pone.0265861;  DOI: 10.1016/j.ejon.2014.02.004).  All overlapping text outside the Methods section has been thoroughly rephrased to ensure originality of expression. Where relevant, appropriate citations to prior work (including our own) have been added. We confirm that the revised manuscript now complies with PLOS ONE’s requirements regarding originality and appropriate citation. |
| 3.Thank you for stating the following financial disclosure:“China Scholarship Council (grant numbers 201806170005).”  Please state what role the funders took in the study. If the funders had no role, please state: "The funders had no role in study design, data collection and analysis, decision to publish, or preparation of the manuscript."  If this statement is not correct you must amend it as needed.  Please include this amended Role of Funder statement in your cover letter; we will change the online submission form on your behalf. | Thank you for your query regarding the funder's role.  As you suggested, we confirmed that the funder (China Scholarship Council, grant number 201806170005) had no role in the study design, data collection and analysis, decision to publish, or preparation of the manuscript.  We had included this amended Role of Funder statement in the cover letter as requested (Please refer to the revised cover letter). |
| 4.Please note that funding information should not appear in any section or other areas of your manuscript. We will only publish funding information present in the Funding Statement section of the online submission form. Please remove any funding-related text from the manuscript. | Thank you for the clarification. We had removed all funding-related text from the manuscript. |
| 5. In the online submission form you indicate that your data is not available for proprietary reasons and have provided a contact point for accessing this data. Please note that your current contact point is a co-author on this manuscript. According to our Data Policy, the contact point must not be an author on the manuscript and must be an institutional contact, ideally not an individual. Please revise your data statement to a non-author institutional point of contact, such as a data access or ethics committee, and send this to us via return email. Please also include contact information for the third party organization, and please include the full citation of where the data can be found. | Thank you for the guidance regarding our data access statement.  We have revised the Data Availability statement to clearly specify:(i) what data can be shared (e.g. de-identified excerpts, coding framework, and thematic framework matrix),(ii) what data cannot be shared (full transcripts, due to the risk of participant identification),(iii) the institutional mechanism for controlled access, and(iv) the criteria for granting access.  **Location:** Page 56, lines 6-15  Revised text: “The qualitative data generated and analysed during this study are not publicly available due to the sensitive nature of the interview content and the risk of participant identification. De-identified excerpts supporting the findings are included within the manuscript. Additional materials, including the coding framework and thematic framework matrix, may be made available on reasonable request. Requests for access should be directed to the Cicely Saunders Institute (King’s College London) via [palliativecare@kcl.ac.uk] and will be subject to review to ensure that the proposed use is consistent with the original ethical approvals and participant consent. Applicants will be required to provide a clear research proposal and agree to data use conditions designed to protect participant confidentiality.” |
| 6. PLOS requires an ORCID iD for the corresponding author in Editorial Manager on papers submitted after December 6th, 2016. Please ensure that you have an ORCID iD and that it is validated in Editorial Manager. To do this, go to ‘Update my Information’ (in the upper left-hand corner of the main menu), and click on the Fetch/Validate link next to the ORCID field. This will take you to the ORCID site and allow you to create a new iD or authenticate a pre-existing iD in Editorial Manager. | Thank you for the reminder. We had validated the ORCID iD in Editorial Manager by following the provided instructions. |
| 7. Please include your full ethics statement in the ‘Methods’ section of your manuscript file. In your statement, please include the full name of the IRB or ethics committee who approved or waived your study, as well as whether or not you obtained informed written or verbal consent. If consent was waived for your study, please include this information in your statement as well. | We have included a full ethics statement in the Methods section of the revised manuscript (page 11). This now specifies the full names of all approving ethics committees and their corresponding approval numbers. We also clearly state that written informed consent was obtained from all participants prior to participation, and that interviews were audio-recorded with participants’ permission. |
| 8. If the reviewer comments include a recommendation to cite specific previously published works, please review and evaluate these publications to determine whether they are relevant and should be cited. There is no requirement to cite these works unless the editor has indicated otherwise. | We thank the editor for this guidance. We have carefully reviewed the reviewer comments to identify any recommendations to cite additional published works. No specific citations were requested by the reviewers. We have therefore not added further references on this basis, but have ensured that all cited literature in the manuscript is relevant, current, and appropriately supports the study. |
| 9. Please review your reference list to ensure that it is complete and correct. If you have cited papers that have been retracted, please include the rationale for doing so in the manuscript text, or remove these references and replace them with relevant current references. Any changes to the reference list should be mentioned in the rebuttal letter that accompanies your revised manuscript. If you need to cite a retracted article, indicate the article’s retracted status in the References list and also include a citation and full reference for the retraction notice. | Thank you for the reminder. We had carefully reviewed the entire reference list to ensure it was complete, correct, and contained no retracted papers. All references were current and relevant to the study. |
| **Reviewer #1** | **Response to Reviewer #1** |
| Thank you for the opportunity to review this manuscript. There are several novel concepts and ideas that are relevant and practical. The follow are my suggestions, comments and suggestions for clarification/clarity and flow for the reader. | We sincerely thank Reviewer 1 for his/her very helpful and constructive comments. We have subsequently addressed each of them and outlined our changes below. |
| Introduction:  Suggest updating references #5-9 (GOLD and WHO reference are a good starting point).  Suggest updating information/references regarding large data sets reference #10.  Consider including the origin of ‘system cluster’ from published literature to clarify ‘phenomenon’. | We have updated the epidemiological and prevalence data using the most recent sources.  **Location**: Page 4, lines 1–2  In addition, we have replaced earlier methodological references (e.g., Dodd et al., 2004) with more recent work:  **Location**: Page 5, lines 1–2  These updates ensure the manuscript reflects the most current evidence base.  We have expanded the Introduction to clarify the origin and development of the symptom cluster concept.  **Location**: Page 5, lines 5–11  The revised text now reads as follows:  “The most recent expert panel defines a symptom cluster as a stable group of two or more concurrent symptoms with temporal characteristics, which are independent of other clusters and may share underlying mechanisms and/or outcomes. Research on symptom clusters originated in nursing science, with seminal work by Dodd et al., and has since evolved across multiple disciplines. This body of work has generated evidence on cluster composition, prognostic significance for clinical outcomes, and their potential utility in identifying targets for intervention.”  This addition provides appropriate theoretical and historical context. |
| Page 5, line 3. Suggest adding or including Nursing research as the origin of current research. Could also include other areas of research in this area based on current research outside of medical applications (or just state this manuscript/study is only for medical/COPD purposes earlier), such as | We thank Reviewer 1 for this insightful suggestion.  We have explicitly acknowledged the contribution of nursing science.  **Location**: Page 5, lines 8–11  The revised text now read as follows:  “…with seminal work by Dodd et al. (2001) in oncology nursing…” |
| Page 7, lines: 5-9. Clarify how the four (4) questions were developed? | Thank you for this request for clarification.  We have clarified how the research questions were developed.  **Location**: Page 7, lines 10–16  The revised text now reads as follows:  “Our research questions were developed deductively from gaps in the existing symptom cluster literature and inductively from established conceptual frameworks…” |
| Page 8, line 14, suggest incorporating/using consistent term of biographical disruption or biographical theory. | Thank you for this important observation regarding terminological consistency.  We have standardised terminology across the manuscript.  **Location**: Multiple sections  (e.g., Page 9, lines 9–10)  The revised text now reads as follows: All instances now consistently use “biographical disruption”. |
| Page 9, line 6, For consistency, flow and clarity who are the‘Participants’ (patients?) based on department or inclusion criteria. | We have clarified that participants are patients with COPD meeting defined inclusion criteria.  **Location**: Page 11, lines 16;  The revised text reads as follows:  “COPD patients, now referred to as participants were recruited…”  Terminology has been standardised throughout. |
| Page 9, line 8. Clarify or include how TOUS will be used in the study along with the ‘strategy mechanism’ of biographical distribution. | Thank you for this valuable suggestion to clarify the integration of our two theoretical frameworks.  We have strengthened the integration of the two theoretical frameworks.  **Location**: Page 17, lines 11–16;  Page 18, lines 1-2.  The Revised text now reads as follows:  “TOUS provided a structured lens to identify and characterise the multidimensional nature and interrelationships of symptoms within clusters. In parallel, the ‘strategy mechanism’ of biographical disruption informed the interpretive analysis by focusing on how participants understood, responded to, and managed the impact of these symptom clusters on their daily lives and sense of self.…”  We believe this clarifies the complementary roles of each framework. |
| Methods:  Page 10, line 6. Clarify who collected the initial patients or participants criteria: PFT’s, etc. | Thank you for this important request for clarification.  We have revised the Methods section to clearly specify how eligibility was determined and who was responsible for identifying relevant clinical information.  **Location**: Page 12, lines 8-10  Revised text: “Eligibility was confirmed through a brief clinical screening conducted by the treating clinician, which included an assessment of the patient’s ability to understand the study information and participate in an interview. No formal cognitive assessment tool was used; rather, clinicians used their professional judgement to ensure that participants had the capacity to provide informed consent and engage meaningfully in the interview.”  This revision now clarifies that clinical data were used as part of routine care and that clinicians were responsible for identifying patients who met the study inclusion criteria. |
| Page 10, lines 6&7, Clarify and for consistency reconcile ‘participants were recruited from’.....and subheading 2.4 includes Recruitment and sampling for flow. | Thank you for this request for clarification.  We have clarified the role of clinicians in identifying eligible participants.  **Location**: Page 12–13  We believe this improves transparency in recruitment procedures. |
| Page 11, line 1, Clarify the clinician(s), healthcare professionals who identified potential candidates for the study that lead to page 11, line 1, study criteria identification. | Thank you for this request for clarification.  We have revised the recruitment section to more clearly specify: which healthcare professionals identified potential participants; how eligibility criteria were applied; and  how participants were approached.  **Location**: Page 12–13  This revision improves transparency regarding the recruitment process and the role of clinicians in identifying eligible participants. |
| Due to the number of theoretical approaches, types of qualitative approaches/processes (descriptive qualitative approach, analysis, etc.), Consolidated Criteria for Reporting Qualitative Research (COREQ), GOLD guidelines/standards, Semi-structured interview topic guide, Framework approach, NVivo, framework matrix, Quality criteria (brief summary of table) | Thank you for this valuable suggestion.  Thank you for this helpful suggestion. We agree that the number of methodological approaches, frameworks, and tools used in the study would benefit from clearer organization.  To address this, we have added a new file summarising the key methodological components of the study and their specific roles.  **Location**: See Supplementary File 2  The added text now reads:  “Overview of methodological approaches and their role in the study, a file (See Supplementary File 2) provides a clear and concise overview of how the study design, data collection methods, analytical approach, theoretical frameworks, and quality criteria fit together, thereby improving clarity and readability.” |
| Data collection:  Page 11. Was a method of triangulation or a similar approach utilized to support or clarify a participant's responses. | Thank you for this important question regarding methodological rigor.  Thank you for this important question regarding methodological rigour.  We confirm that triangulation was employed in this study and have revised the manuscript to clarify this more explicitly.  **Location**: Page 18, lines 4–9  The revised text now reads as follows:  “To enhance the credibility of the findings, we employed triangulation. First, data source triangulation was used by comparing participants’ interview accounts with relevant clinical information (e.g., GOLD stage, spirometry results) to contextualise symptom experiences. Second, analyst triangulation was undertaken, whereby two researchers independently coded the data and discussed emerging interpretations, with discrepancies resolved through discussion with a third researcher. This process strengthened the rigour and trustworthiness of the analysis.” |
| Page 14, Clarify how ‘strategy mechanism’ of biographical distribution used in addition to or in combination with TOUS- i can see/read this is mentioned in Table 2, suggest mentioning this earlier in this section as only TOUS seems to be mentioned. | Thank you for this insightful observation. We agree that the relationship between the two theoretical frameworks required clearer articulation earlier in the Data Analysis section.  We have revised the manuscript to introduce and integrate both frameworks at the outset of the analysis, rather than only referring to biographical disruption later (e.g., in Table 2).  **Location**: Page 17, lines 11–16  Page 18, lines 1–2  Revised text now reads as follows:  “The analysis was informed by two complementary theoretical frameworks: the Theory of Unpleasant Symptoms (TOUS) and the concept of biographical disruption. TOUS provided a structured lens to identify and characterise the multidimensional nature and interrelationships of symptoms within clusters. In parallel, the ‘strategy mechanism’ of biographical disruption informed the interpretive analysis by focusing on how participants understood, responded to, and managed the impact of these symptom clusters on their daily lives and sense of self. The combined use of these frameworks enabled both descriptive and interpretive depth in the analysis.”  We believe this revision clarifies the distinct and complementary roles of each framework, with TOUS informing the identification and characterisation of symptom clusters, and biographical disruption guiding the interpretation of how participants understood and responded to these experiences. |
| Data analysis:  Page 15, line 9, Table 2, consider incorporating how ‘strategy mechanism’ of biographical distribution and TOUS aided in the rigor of the analysis. | Thank you for this valuable suggestion.  We agree that the role of the theoretical frameworks in supporting analytical rigour required clearer articulation. We have revised Table 2 and the Data Analysis section to explicitly describe how the Theory of Unpleasant Symptoms (TOUS) and the concept of biographical disruption contributed to the rigour of the analysis.  **Locations:**  Page 18, lines 3-4 (Data Analysis section)  Table 2  Revised text:  “The use of TOUS and the concept of biographical disruption contributed to analytical rigour by providing theoretically informed frameworks that supported consistent and conceptually grounded interpretation…”  Add to Table 2  “Theoretical integration - Use of TOUS and biographical disruption frameworks - Provided a structured and conceptually grounded lens for data interpretation, enhancing analytical depth, consistency, and transparency in linking data to themes.”    These revisions clarify how the frameworks enhanced the depth, coherence, and transparency of the analysis.” |
| Discussion:  For Discussion section and the entire manuscript- in addressing frontline healthcare providers/professionals, all health care professionals contributing and working for the COPD patients/clients in all settings including ‘Respiratory Nursing’. In addition, patient care settings, -hospital, clinic, subacute, long-term rehabilitation, cardiopulmonary rehabilitation, And healthcare professionals, physicians, medical training, respiratory therapist, emergency department personnel, clinics, home care assistants where nursing aids, medical assistants, etc. These healthcare professionals may be the first to see ‘system clusters’ (signs and symptoms) and ‘triggered chain reaction in addition to the setting where they are first seen. | Thank you for this important and helpful suggestion. We agree that the relevance of our findings extends beyond a single professional group or care setting. We have revised the Discussion to explicitly reflect the multidisciplinary nature of COPD care and the range of settings in which symptom clusters and ‘trigger’ symptoms may first be identified.  **Location**: Page 52, lines 5–16  The revised text reads:  “the identification and management of symptom clusters and their potential ‘trigger’ symptoms are not confined to a single professional group or care setting. Patients with COPD interact with a wide range of healthcare professionals across the care pathway, including physicians, nurses, respiratory therapists, emergency department staff, rehabilitation teams, and community-based practitioners. These professionals, working in diverse settings such as hospitals, outpatient clinics, subacute and long-term care facilities, cardiopulmonary rehabilitation services, and home care environments, may all be the first to recognise symptom clusters and their dynamic interrelationships. Our findings therefore highlight the need for a multidisciplinary approach, in which all healthcare professionals are supported to recognise, assess, and respond to symptom clusters and ‘trigger’ symptoms as part of routine care. This includes ensuring that education, training, and clinical systems facilitate early identification and coordinated management across settings.”  In addition, we have ensured consistent use of the term “healthcare professionals” throughout the manuscript where appropriate. This revision strengthens the clinical applicability of our findings across the full care pathway. |
| ‘Symptoms clusters’ and trigger-based' should be added as a part of patient assessment/physical exam for these patients/clients. | Thank you for this valuable suggestion.  We have revised the Discussion to explicitly emphasise that the assessment of symptom clusters and ‘trigger’ symptoms should be incorporated into routine clinical assessment and physical examination.  **Location**: Page 51, lines 9–14  The revised text reads as follows:  “Assessment of symptom clusters and identification of ‘trigger’ symptoms should be incorporated as standard components of routine clinical assessment and physical examination for individuals with COPD. Rather than assessing symptoms in isolation, healthcare professionals should actively explore how symptoms co-occur, interact, and potentially trigger one another. This approach may enable earlier recognition of deteriorating symptom patterns and support more targeted and coordinated symptom management. “  We believe this revised text strengthens the clinical applicability of our findings and highlights their relevance for routine practice. |
| Conclusion:  Consider another possible reason for declining the interviews might be time as the interviews were conducted following treatment, too tired, no time. | Thank you for this helpful suggestion.  We have revised the limitations section to acknowledge that some eligible participants may have declined participation due to practical constraints such as time limitations or fatigue following treatment.  **Location**: Page 50, lines 10–13  The revised text reads: In addition, some eligible participants may have declined to take part due to practical constraints, including limited time availability or fatigue following treatment, which may have influenced participation and the range of perspectives captured.  This addition improves transparency regarding potential factors influencing recruitment and participation. |
| Add/incorporate ‘trigger’ concept along with ‘symptom clusters’ in manuscript for clarity and consistency with title - examples: in Figures 2, 3, | Thank you for this helpful suggestion. We agree that the concept of ‘trigger’ symptoms required clearer and more consistent integration throughout the manuscript.  We have made the following revisions:  Introduced a clear definition of ‘trigger’ symptoms in the Results section;  Ensured consistent use of the term alongside “symptom clusters” in key sections of the manuscript;  Revised Figures 3 to explicitly emphasize the role of ‘trigger-based symptom cluster”  **Location**s:  Definition added: Page 23, lines 10–12  Figures updated: Figures 3  Revised text (example):  “In this study, ‘trigger’ symptoms refer to symptoms that appear to initiate or exacerbate a cascade of interconnected symptoms…”  These revisions improve conceptual clarity and ensure alignment between the title, text, and figures. We believe these revisions improve conceptual clarity and ensure alignment between the title, text, and figures. |
| Figure 1., clarify 2- ’need to do a temporary physical examination’ | Thank you for this helpful comment. We agree that the wording in Figure 1 was unclear. We have revised the text to improve clarity and align with standard clinical terminology.  **Location**: Figure 1  Revised text:  “need to do a temporary physical examination (e.g.X-ray test, blood routine analysis)  This revision now improves clarity and ensures appropriate clinical phrasing. |
| Clarify how and if physical assessment/physical exam use of ‘signs’ (signs and symptoms) are part of or not part of this approach when objective data/finding of PFT information is used in selection criteria. | Thank you for this important request for clarification.  We have revised the manuscript to clearly distinguish the role of objective clinical data and physical assessment findings from the qualitative analysis.  **Location**: Page 18, lines 12–16  The revised text reads:  “Objective clinical data (e.g., pulmonary function test results, GOLD classification) were used solely to confirm eligibility and to provide clinical context for the sample. The qualitative analysis focused on participants’ subjective experiences of symptoms as described in interviews. Clinical signs and findings from physical examination were not analysed as data within this study but formed part of the broader clinical background in which participants’ experiences were situated.”  This clarification ensures that it is clear that the study findings are based on participants’ subjective symptom experiences, while objective clinical data were used only for eligibility and contextual purposes. |
| **Reviewer #2** | **Response to Reviewer #2** |
| I appreciate you submitting this timely qualitative paper. The topic is clinically meaningful, and the manuscript is generally well organized and easy to follow. I appreciate the effort to report the work using COREQ and to present a clear thematic structure.  To move the paper forward, I recommend minor revision, focused on a small set of required reporting and compliance clarifications (no additional data collection is requested): | We thank Reviewer 2 for his/her very helpful and constructive comments. |
| A- Replace any “XX” placeholders with the correct approval number(s) and approving bodies, and ensure the ethics information is consistent across the manuscript and submission fields. | Thank you for highlighting this important issue.  We have carefully reviewed the manuscript and replaced all placeholder text (e.g., “XX”) with the correct ethics approval details. We have also ensured that the ethics information is fully consistent across the manuscript and submission materials.  **Location**: Page 11, lines 7–13  The revised text now reads as follows:  “Ethical approval for this study was obtained from researcher’s institutional review board (approval number: HR-18/19- 13608), and also obtained ethical approvals from the Second People’s Hospital of Changzhou Ethics Committee (approval number: [2019] KY063-01), the Affiliated Huai’an No.1 People's Hospital of Nanjing Medical University Ethics Committee (approval number: KY-P-2019-048-01) and the Affiliated Huai’an Hospital of Xuzhou Medical University Ethics Committee (approval number: HEYLL201932).” |
| B- The manuscript refers to both written and verbal consent. Please clarify clearly whether consent was written, verbal, or both—and specify what each covered (participation, audio-recording, access to records if applicable). Make sure the description is consistent across all sections. | Thank you for this important comment. We have revised the manuscript to clearly and consistently describe the consent procedures used in the study.  Written informed consent was obtained from all participants prior to participation. This included consent to take part in the study, to audio-record interviews, and to use anonymised data for research purposes. Verbal consent was additionally confirmed at the start of each interview to ensure participants’ ongoing willingness to participate.  We have ensured that this description is consistent across all relevant sections of the manuscript.  **Location**: Page 14, lines 6–10  Revised text: “Written informed consent was obtained from all participants prior to participation. This included consent to take part in the study, to audio-record the interviews, and to use anonymised data for research purposes. Verbal consent was additionally confirmed at the start of each interview to ensure participants’ ongoing willingness to participate.” |
| C- As you know, qualitative transcripts may reasonably be restricted for privacy reasons; however, the Data Availability statement should explain the access route more precisely than “upon request.” Please revise the statement to specify what can be shared (e.g., de-identified excerpts, codebook/framework matrix) and, if transcripts are restricted, provide a clear controlled-access mechanism (e.g., institutional/ethics contact and criteria for access). | Thank you for this important comment regarding data transparency. We have revised the Data Availability statement to clearly specify:_(i) what data can be shared (e.g. de-identified excerpts, coding framework, and thematic framework matrix),_(ii) what data cannot be shared (full transcripts, due to the risk of participant identification),_(iii) the institutional mechanism for controlled access, and_(iv) the criteria for granting access.  **Location:** Page 56, lines 7-16  Revised text: “The qualitative data generated and analysed during this study are not publicly available due to the sensitive nature of the interview content and the risk of participant identification. De-identified excerpts supporting the findings are included within the manuscript. Additional materials, including the coding framework and thematic framework matrix, may be made available on reasonable request. Requests for access should be directed to the Cicely Saunders Institute (King’s College London) via [palliativecare@kcl.ac.uk] and will be subject to review to ensure that the proposed use is consistent with the original ethical approvals and participant consent. Applicants will be required to provide a clear research proposal and agree to data use conditions designed to protect participant confidentiality.” |
| D- Please briefly explain how the purposive sampling plan was operationalized (how participants were approached/selected) and clarify what is meant by “clinical interview and cognitive assessment” (what tool or process was used and how it informed eligibility). | Thank you for this important request for clarification.  We have revised the Methods section to more clearly describe how purposive sampling was operationalised and to clarify the process referred to as “clinical interview and cognitive assessment.”  Specifically:  We now explain how clinicians identified and approached eligible participants during routine clinical care and how purposive sampling was used to ensure diversity in participant characteristics  We have clarified that eligibility included a brief clinical screening conducted by clinicians to assess participants’ ability to provide informed consent and participate in the interview, and that no formal cognitive assessment tool was used  **Location**: Page 12, lines 8–11  Revised text: “Eligibility was confirmed through a brief clinical screening conducted by the treating clinician, which included an assessment of the patient’s ability to understand the study information and participate in an interview. No formal cognitive assessment tool was used; rather, clinicians used their professional judgement to ensure that participants had the capacity to provide informed consent and engage meaningfully in the interview.”  These revisions improve transparency and ensure clarity regarding recruitment and eligibility procedures. |
| E- Please add a short note on transcription accuracy checks and how translation of quotes/extracts was handled to preserve meaning. | Thank you for this important methodological clarification.  We have revised the manuscript to clarify the processes used to ensure transcription accuracy and, where applicable, the handling of translation.  **Location**: Page 17, lines 5–9  Revised text: “All interviews were audio-recorded and transcribed verbatim. To ensure accuracy, transcripts were checked against the original audio recordings by the research team. Where interviews or excerpts were translated into English, translation was undertaken by bilingual members of the research team. Translated excerpts were reviewed to ensure that the original meaning and context were preserved.”  This addition strengthens transparency regarding data quality and the preservation of meaning in the analysis. |

Once again, thank you very much for your time in considering our manuscript for publication in PLOS One. We look forward to hearing from you.

With all good wishes.

**References**

Bury M. Chronic illness as biographical disruption. Sociol Health Illn. 1982;4(2):167-182. doi: 10.1111/1467-9566.ep11339939

Dodd MJ, Miaskowski C, Lee KA. Occurrence of symptom clusters. JNCI Monogr. 2004;(32):76-78. doi: 10.1093/jncimonographs/lgh008

Harris CS, Dodd M, Kober KM, Dhruva AA, Hammer MJ, Conley YP, Miaskowski CA. Advances in Conceptual and Methodological Issues in Symptom Cluster Research: A 20-Year Perspective. ANS Adv Nurs Sci. 2022 Oct-Dec;45(4):309-322. doi: 10.1097/ANS.0000000000000423. Epub 2022 Apr 28. PMID: 35502915; PMCID: PMC9616968
